# Dosimetry of small bone joint calculated by the analytical anisotropic algorithm: a Monte Carlo evaluation using the EGSnrc

**DOI:** 10.1120/jacmp.v15i1.4588

**Published:** 2013-01-06

**Authors:** James C. L. Chow, Runqing Jiang, Amir M. Owrangi

**Affiliations:** ^1^ Department of Radiation Oncology Princess Margaret Caner Center, University Health Network Toronto ON Canada; ^2^ University of Toronto and Radiation Medicine Program Princess Margaret Caner Center, University Health Network Toronto ON Canada; ^3^ Medical Physics Department Grand River Regional Cancer Center Kitchener ON Canada; ^4^ Physics Department University of Waterloo Waterloo ON Canada; ^5^ Department of Radiation Oncology University of Michigan Health System Ann Arbor MI USA

**Keywords:** bone heterogeneity, heterogeneous correction, anisotropic analytical algorithm, dose calculation

## Abstract

This study compared a small bone joint dosimetry calculated by the anisotropic analytical algorithm (AAA) and Monte Carlo simulation using megavoltage (MV) photon beams. The performance of the AAA in the joint dose calculation was evaluated using Monte Carlo simulation, and dependences of joint dose on its width and beam angle were investigated. Small bone joint phantoms containing a vertical water layer (0.5‐2 mm) sandwiched by two bones ($2\times 2\times 2\;{\text{cm}}^{3}$) were irradiated by the 6 and 15 MV photon beams with field size equal to $4\times 4\;{\text{cm}}^{2}$. Depth doses along the central beam axis in a joint (cartilage) were calculated with and without a bolus (thickness=1.5cm) added on top of the phantoms. Different beam angles (0°‐15°) were used with the isocenter set to the center of the bone joint for dose calculations using the AAA (Eclipse treatment planning system) and Monte Carlo simulation (the EGSnrc code). For dosimetry comparison and normalization, dose calculations were repeated in homogeneous water phantoms with the bone substituted by water. Comparing the calculated dosimetry between the AAA and Monte Carlo simulation, the AAA underestimated joint doses varying with its widths by about 6%‐12% for 6 MV and 12%‐23% for 15 MV without bolus, and by 7% for 6 MV and 13%‐17% for 15 MV with bolus. Moreover, joint doses calculated by the AAA did not vary with the joint width and beam angle. From Monte Carlo results, there was a decrease in the calculated joint dose as the joint width increased, and a slight decrease as the beam angle increased. When bolus was added to the phantom, it was found that variations of joint dose with its width and beam angle became less significant for the 6 MV photon beams. In conclusion, dosimetry deviation in small bone joint calculated by the AAA and Monte Carlo simulation was studied using the 6 and 15 MV photon beam. The AAA could not predict variations of joint dose with its width and beam angle, which were predicted by the Monte Carlo simulations.

PACS numbers: 87.55.K‐; 87.53.Bn; 87.53.‐j

## I. INTRODUCTION

In radiotherapy involving a bone joint or interface, dose enhancement at the bone cartilage is expected due to surrounding bone scatter.^1^, ^2^, ^3^, ^4^ As the cartilage is a soft flexible connective tissue constructed for patient's movement, an accurate dose calculation for this thin tissue layer is needed. This is because stiff joint is a typical side effect in radiotherapy.^5^, ^6^, ^7^ The typical anatomy of a bone joint contains a thin soft tissue with thickness in millimeter sandwiched by two bones. As the electron density per unit volume of bone is higher than soft tissue or water, extra dose from bone scatter contributes to the joint when using megavoltage (MV) photon beams.^8^, ^9^

In external beam treatment planning, the superposition/convolution method is commonly used in dose calculation and the accuracy has been verified using different heterogeneous phantoms.^10^, ^11^, ^12^, ^13^ The anisotropic analytical algorithm (AAA) uses a pencil beam modeled by Monte Carlo simulation and adjusted from measurement to consider primary photons, scattered extrafocal photons and scattered electrons.^14^, ^15^ In the AAA, the dose distribution in the lateral direction of the pencil beam is scaled by the equivalent path length to the calculation point in the previous volume layer, while the dose distribution in the longitudinal direction is scaled by the equivalent path length. The AAA, therefore, considers the tissue heterogeneity using lateral scaling in a spherical plane normal to the propagation direction of the pencil beam. If the irradiated target is a bone joint with the pencil beam parallel to the cartilage surface, the scaling plan perpendicular to the pencil beam includes the bone heterogeneity and thin soft tissue layer at the joint. Since the AAA only uses an approximate direct particle transport model for the electron transport, there would be uncertainty in charge particle and photon scatter equilibrium in dose calculation when the geometry is a very thin soft tissue (millimeter scale) sandwiched by two bone heterogeneities with higher relative electron density.

Monte Carlo simulation provides a possible way to predict the bone joint dose within small heterogeneities (e.g., tissue‐bone interface). Monte Carlo methods perform a step‐by‐step charge/uncharged particle transport in various media with different morphologies. Unlike other commercial semiempirical/analytic dose calculation algorithm such as superposition/convolution, Monte Carlo simulation is independent of the assumption of electronic equilibrium and, therefore, provides with a higher degree of accuracy compared to other algorithms when predicting dose deposited by photon beams.^(16)^ Moreover, Monte Carlo methods have been well known as a bench mark in predicting dosimetry in a heterogeneous system with tissue, air, and bone, though dose calculation takes a longer time than the more practical superposition/convolution method in treatment planning.^17^, ^18^, ^19^

The aim of this study is to evaluate the performance of the AAA to calculate the dose in a small bone joint (e.g., finger and toe) using the Monte Carlo simulations. The deviations of depth doses along the cartilage sandwiched by bones were found by comparing results predicted between the AAA and the Monte Carlo simulation. Moreover, dependences of the joint dose on its width and the beam angle were investigated. In this study, Monte Carlo simulations were carried out using the EGSnrc‐based codes.^(20)^

## II. MATERIALS AND METHODS

### A. Phantom and beam geometry

**Figure 1:**
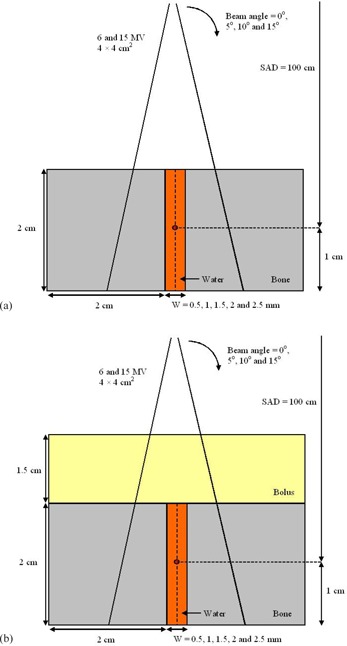
Schematic diagrams (not to scale) showing the bone joint phantoms (a) without and (b) with a bolus of 1.5 cm. The thickness of the vertical water layer, mimicking the cartilage or bone joint, is equal to 0.5—2.5 mm using the 6 and 15 MV photon beams with field size of $4\times 4\;{\text{cm}}^{2}$.

Two groups of small heterogeneous phantoms containing bone and water were used in this study. The first group of phantoms, as shown in Fig. 1(a), had a thin water layer (bone joint or cartilage) vertically sandwiched by two bones. The cartilage was assumed to be water‐equivalent. The joint width was varied from 0.5 to 2.5 mm and the dimension of the two bones was $2\times 2\times 2\;{\text{cm}}^{3}$. This phantom geometry mimics a finger joint irradiated by the photon beams. Both 6 and 15 MV photon beams with field size of $4\times 4\;{\text{cm}}^{2}$ were used to irradiate a joint with the isocenter set in the middle of water layer (Fig. 1(a)). The source‐to‐axis distance was equal to 100 cm. Apart from irradiation at a beam angle of 0°, the photon beam was rotated to 5°, 10°, and 15° based on our clinical experience. Depth doses along the cartilage (vertical broken line in Fig. 1(a)) were calculated with different joint widths, beam angles, and energies in the phantoms. This phantom geometry can evaluate the dose calculation performance of the AAA in the buildup region with bone scatter varied by different joint widths.

Since bolus was usually used in the joint irradiation clinically, another group of phantoms was designed the same as Fig. 1(a), except a bolus layer of 1.5 cm was added as seen in Fig. 1(b). With the addition of bolus, depth doses beyond the buildup region of the 6 MV photon beams can be calculated and compared. The beam geometry and energy in Figs. 1(a) and 1(b) are the same.

### B. Dose calculations

*B.1 Treatment planning system using the AAA*

Bone joint phantoms, as shown in Figs. 1(a) and 1(b), were created using the Eclipse treatment planning system (version 10.0.28, Varian Medical Systems, Palo Alto, CA). The bone density was set to 1.75 g cm^−3^, the same as in Monte Carlo simulation (ICRP 1975).^(21)^ Photon beams of 6 and 15 MV ($\text{field size}=4\times 4\;{\text{cm}}^{2}$) produced by the Varian 21 EX linear accelerator were used in the irradiation. The dose grid was set to the minimum of 1 mm, according to the treatment planning system.^(22)^ Depth doses along the cartilage (vertical broken line in Figs. 1(a) and 1(b)) were calculated for different joint widths of 0.5, 1, 1.5, 2, and 2.5 mm and beam angles of 0°, 5°, 10°, and 15°. All regions outside the phantom were set to air as default in the dose calculation. For dosimetric comparison and normalization, all dose calculations were repeated in homogeneous water phantoms with the bones in Fig. 1 replaced by water.

#### B.2 Monte Carlo simulation using the EGSnrc

Phase space files of the 6 and 15 MV beams with field size equal to $4\times 4\;{\text{cm}}^{2}$ produced by the Varian 21 EX linear accelerator were generated using the BEAMnrc code.^23^, ^24^ Verifications of phase space files by measurements using ionization chambers, done elsewhere,^(21)^ were not presented here. Phantoms and beam geometries as shown in Fig. 1 were input to the DOSXYZnrc for dose calculations using the 6 and 15 MV beams.^25^, ^26^ ICRPBONE700ICRU and H2O700ICRU were selected as the bone and joint tissue (water‐equivalent) materials in simulations. The voxel size was set to $0.5\times 0.5\times 0.5\;{\text{mm}}^{3}$ corresponding to the x‐, y‐, and z‐axes. Depth doses along the vertical broken lines in Fig. 1 were calculated per beam geometries in the figure. The electron and photon cutoff energy was set to 700 keV and 10 keV, respectively. Five hundred million histories were run in each calculation. With this number of histories, the relative dose error of the statistical uncertainty as a fraction of the dose in voxel was within ±1% according to our EGSnrc dose output files.^(26)^ Monte Carlo simulations were repeated in homogeneous water phantoms with the bones in Fig. 1 replaced by water, using the same beam energy and geometry.

## III. RESULTS

**Figure 2:**
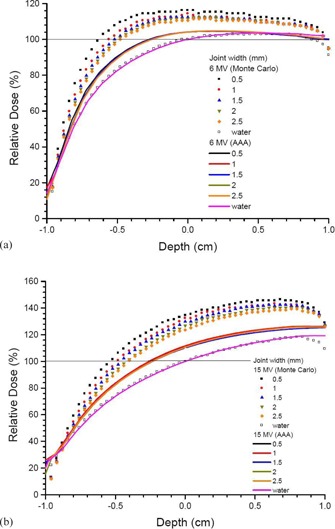
Relative depth doses of bone joint phantoms calculated along the vertical broken line of Fig. 1(a) using the (a) 6 and (b) 15 MV photon beams. The AAA and Monte Carlo simulation were used in dose calculations with the joint width varying from 0.5—2.5 mm. All depth doses in the bone joint phantoms were normalized to the dose at the isocenter of a homogeneous phantom with the bone replaced by water (Fig. 1(a)). A horizontal line of 100% dose is added to guide the normalization.

**Figure 3:**
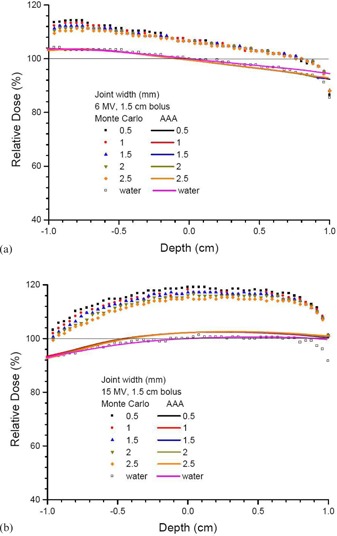
Relative depth doses of bone joint phantoms calculated along the vertical broken line of Fig. 1(b) using the (a) 6 and (b) 15 MV photon beams. The AAA and Monte Carlo simulation were used in dose calculations with the joint width varying from 0.5—2.5 mm. All depth doses in the bone joint phantoms were normalized to the dose at the isocenter of a homogeneous phantom with the bone replaced by water (Fig. 1(a)). The thickness of bolus was equal to 1.5 cm. A horizontal line of 100% dose is added to guide the normalization.

**Figure 4:**
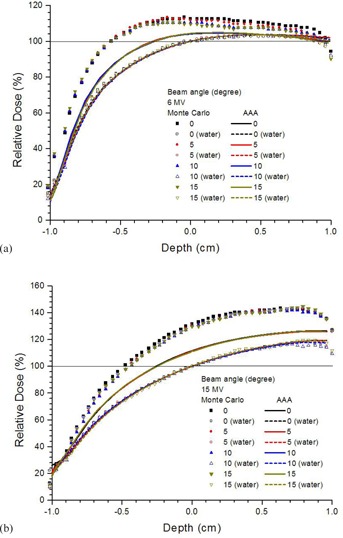
Relative depth doses of the bone joint phantom (Fig. 1(a)) with the joint width equal to 1 mm using the (a) 6 and (b) 15 MV photon beams. The AAA and Monte Carlo simulation were used to calculate the dose with the beam angle varied from 0° to 15°. All depth doses of the bone joint phantom were normalized to the isocenter of the corresponding water phantom with the bone (Fig. 1(a)) replaced by water. Relative depth doses of water phantoms with the same dimension of the bone joint phantoms are also shown for comparison. A horizontal line of 100% dose is added to guide the normalization.

**Figure 5:**
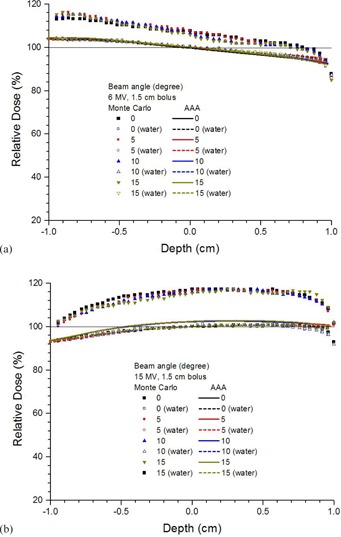
Relative depth doses of the bone joint phantom (Fig. 1(b)) with the joint width equal to 1 mm using the (a) 6 and (b) 15 MV photon beams. The AAA and Monte Carlo simulation were used to calculate the dose with the beam angle varied from 0° to 15°. All depth doses of the bone joint phantom were normalized to the isocenter of the corresponding water phantom with the bone in Fig. 1(a) replaced by water. Relative depth doses of the water phantom with the same dimension of the bone joint phantom are also shown for comparison. A horizontal line of 100% dose is added to guide the normalization.

Depth doses of a small bone joint as shown in Fig. 1(a) are shown in Figs. 2(a) and 2(b) using the 6 and 15 MV photon beams, respectively. Both results calculated by the AAA and Monte Carlo simulations are shown with joint widths equal to 0.5, 1, 1.5, 2, and 2.5 mm. Depth doses of homogeneous water phantoms with the same dimension of phantoms and beam configurations as in Fig. 1(a) are also shown in Fig. 2. For bone joint phantoms with bolus (Fig. 1(b)), depth doses with the same range of joint widths and beam geometries in Fig. 2 are shown in Fig. 3(a) (6 MV) and Fig. 3(b) (15 MV). In Figs. 2 and 3, all depth doses were normalized to the dose at isocenter of the corresponding water phantom in the figure. Therefore, all depth doses were related to the dose at the isocenter of a homogeneous water phantom. For the effect of beam angle on the depth dose in a joint, Figs. 4(a) and 4(b) show depth doses of a bone joint (Fig. 1(a)) with beam angles equal to 0°, 5°, 10°, and 15° using the 6 and 15 MV photon beams. Depth doses of corresponding water phantoms (bone replaced by water) are also shown in Fig. 4 for comparison. Depth doses using the same beam geometry of Fig. 4 but different phantoms as seen in Fig. 1(b) are shown in Fig. 5. Same as Figs. 2 and 3, depth doses of heterogeneous phantoms in Figs. 4 and 5 were normalized to the dose at isocenter in corresponding water phantoms. To compare the dose at a bone joint, relative doses at the isocenter of phantoms (Figs 1(a) and 1(b)) with different joint widths and beam angles are shown in Table 1. Discrepancies between results from the AAA and Monte Carlo simulation are also shown in Table 1, with negative value reflecting dosimetric underestimation by the AAA. All doses at the isocenters of bone joint phantoms were normalized to doses at the isocenters of corresponding water phantoms.

**Table 1:** Relative doses and discrepancies between the AAA and Monte Carlo simulation at the isocenters of the bone joint phantoms (with and without bolus) with joint width varying from 0.5‐2.5 mm, and with joint width equal to 1 mm and the beam angle varied from 0° to 15°. All doses were calculated using the AAA and Monte Carlo simulation and normalized to doses at isocenters of homogeneous phantoms with water substituted for the bone and with the same beam geometry (Fig. 1). Negative value of discrepancy reflects the dosimetric underestimation by the AAA

	*No Bolus*						*Bolus*					
*Joint Width*	*Monte Carlo (%)*		*AAA (%)*		*Discrepancy (%)*		*Monte Carlo (%)*		*AAA (%)*		*Discrepancy (%) (mm)*	
	*6 MV*	*15 MV*	*6 MV*	*15 MV*	*6 MV*	*15 MV*	*6 MV*	*15 MV*	*6 MV*	*15 MV*	*6 MV*	*15 MV*
0.5	116.2	134.7	104.1	111.6	‐12.1	‐23.1	106.6	119.1	99.6	102.2	‐7.0	‐16.9
1.0	114.4	132.5	104.1	111.8	‐10.3	‐20.7	106.6	118.2	99.6	102.2	‐7.0	‐16.0
1.5	112.4	129.9	104.1	111.4	‐8.3	‐18.5	106.6	117.2	99.5	102.2	‐7.1	‐15.0
2.0	111.4	126.8	104.1	111.5	‐7.3	‐15.3	106.6	116.1	99.5	102.2	‐7.1	‐13.9
2.5	110.5	123.6	104.1	111.6	−6.4	−12.0	106.7	115.3	99.5	102.2	−7.2	−13.1
	*No Bolus*						*Bolus*					
*Beam Angle (degree)*	*Monte Carlo (%)*		*AAA (%)*		*Discrepancy (%)*		*Monte Carlo (%)*		*AAA (%)*		*Discrepancy (%) (mm)*	
	*6 MV*	*15 MV*	*6 MV*	*15 MV*	*6 MV*	*15 MV*	*6 MV*	*15 MV*	*6 MV*	*15 MV*	*6 MV*	*15 MV*
0	112.4	131.5	104.0	111.6	−8.4	−19.9	108.6	117.2	100.3	102.3	−8.3	−14.9
5	111.6	130.9	104.1	111.3	−7.5	−19.6	107.6	116.5	100.1	102.4	−7.5	−14.1
10	110.4	130.3	104.0	111.5	−6.4	−18.8	106.7	115.9	100.0	102.2	−6.7	−13.7
15	109.4	129.7	104.3	111.5	−5.1	−18.2	105.7	115.4	100.1	102.4	−5.6	−13.0

## IV. DISCUSSION

### A. Depth dose dependence on the joint width

Effect of the bone scatter varying with the joint width on depth dose can be seen in Fig. 2. Since no bolus was used, a bone joint was irradiated in the buildup region of the photon beams. It is seen in Figs. 2(a) and 2(b) that depth doses calculated by Monte Carlo simulations agreed well with those calculated by the AAA in water phantoms for the 6 and 15 MV photon beams. The only significant deviation was the exit doses in the bottom of phantoms (close to 1 cm in Fig. 2), where Monte Carlo results were lower than the AAA. This reflects that dose calculation using the AAA was performed well, except when handling the exit dose in the water phantom. In calculating the exit dose, the AAA neglected the loss of dose caused by the backscatter near the bottom of phantom interface. For bone joint phantoms as shown in Fig. 1(a), bone dose enhancement can be found in both depth doses at the joint calculated by the AAA and Monte Carlo simulation. However, it is seen in Fig. 2 that depth doses calculated by the AAA did not vary with the joint width as calculated by Monte Carlo simulation. In Monte Carlo simulation, depth dose increased with a decrease of joint width. As a result of the proximity of the bone to the central axis, more bone scatter contributed to the depth dose. Moreover, the calculated bone dose enhancement for the AAA was lower than Monte Carlo simulation, and the depths of maximum dose for the bone joint phantoms were smaller than those for the water phantoms.

Figure 3 shows depth doses with a bolus on top of the bone joint phantom. The thickness of the bolus (1.5 cm) was equal to the depth of maximum dose of the 6 MV photon beams. In this experimental configuration, the effect of joint width on the depth dose beyond the buildup region of the 6 MV photon beams could be seen. As for the nonbolus results shown in Fig. 2, depth doses in water calculated by the AAA and Monte Carlo simulation agreed well, except for the exit dose due to the disregard of the backscatter near the bottom of phantom by the AAA. Beyond the buildup region of the 6 MV photon beams (Fig. 3(a)), deviations between the bone joint and water depth doses were less significant than in the buildup region. This may be due to the missing of bone scatter caused by the bolus volume (Fig. 1(b)) which was replaced by the bone in Fig. 1(a). Moreover, the bone dose enhancement calculated by the AAA was much less than that by Monte Carlo simulation. Variation of depth dose with joint width was found to be less significant in the 6 MV photon beams when comparing Figs. 3(a) and 3(b). This is due to the shorter secondary electron path of the 6 MV photon beams compared to 15 MV, and depth doses (Fig. 3(a)) of 6 MV beams calculated beyond the buildup region. It is also found that depth doses calculated by the AAA did not vary with the joint width, compared to Monte Carlo results. The AAA uses a beam and patient geometry based on divergent beamlets. The effect of this approach and the essential pencil beam nature of the algorithm showed that the AAA did not model the dosimetry well in the current geometries.

### B. Depth dose dependence on the beam angle

Effect of the beam angle on depth dose in bone joint phantoms can be seen in Figs. 4 and 5 for the 6 and 15 MV photon beams. Beam angles of 0°, 5°, 10°, and 15° were used in the bone joint phantoms and homogeneous water phantoms. In the buildup region with joint width equal to 1 mm, it is seen in Fig. 4(a) that the depth of maximum dose decreased when the bone was present in the phantom. Moreover, depth dose below the isocenter of the phantom decreased with an increase of beam angle when dose was calculated by Monte Carlo simulation using the 6 MV photon beams. However, depth dose did not vary with the beam angle when calculated by the AAA in both the heterogeneous and homogeneous phantoms. The bone dose enhancement calculated by the AAA was less than that calculated by Monte Carlo simulation. When a bolus of 1.5 cm was added to the bone joint phantom, the bone dose enhancement calculated by the AAA decreased compared to the phantom without bolus due to the lack of bone scatters from the bolus in Fig. 1(b). In Figs. 4 and 5, the AAA again did not model the exit dose of the phantom compared to Monte Carlo simulation. Comparing to Monte Caro results, it is found that the AAA underestimated the depth dose at a bone joint with variation of beam angle.

### C. Dosimetric variations at a bone joint

To study the deviation of dose calculated by the AAA and Monte Carlo simulation quantitatively, dose at the isocenter in a bone joint calculated by both methods are compared in Table 1. All doses in Table 1 were normalized to doses at the isocenters in water phantoms with the same dimension of the corresponding bone joint phantoms (Fig. 1). Without using bolus, it is seen in Table 1 that the dose calculated by Monte Carlo simulation at the bone joint decreased with an increase of the joint width. The dose decreased from 116.2% to 110.5% and 134.7% to 123.6% for the 6 and 15 MV, respectively. However, doses at the same point calculated by the AAA did not vary significantly with the joint width. When bolus was added to the phantom, the bone joint dose was found to vary more significantly with the joint width for the 15 MV photon beam compared to 6 MV. This shows that beyond the buildup region, effect of joint width was insignificant in the joint dose for the 6 MV photon beams. However, joint width is still found to affect the joint dose for the 15 MV photon beams due to the relatively longer secondary electron path. Compared to Monte Carlo results, it is seen in Table 1 that the AAA underestimated joint doses to 7% for 6 MV and 13%‐17% for 15 MV with bolus, and 6%‐12% for 6 MV and 12%‐23% for 15 MV without bolus on the phantoms. Variation of joint dose on the beam angle is also reported in Table 1, using the same normalization method as above. Monte Carlo results show that with an increase of photon beam angle from 0° to 15°, the joint dose decreased slightly from 112.4% to 109.4% and 131.5% to 129.7% for the 6 and 15 MV photon beams, respectively, without bolus. This is different from the AAA results using the same beam geometry and phantom, in which joint doses did not vary significantly. When bolus was added, joint dose calculated by Monte Carlo simulation decreased from 108.6% to 105.7% (6 MV) and 117.2% to 115.4% (15 MV) with an increase of beam angle. However, such a decrease of dose could not be predicted by the AAA. Future work includes using a more realistic bone anatomy considering both the cortical (hard) and spongy (soft) bone in the phantom of study.

## V. CONCLUSIONS

Dosimetry in a small bone joint with variations of joint width and beam angle was compared between the AAA and Monte Carlo dose calculation method. From depth doses calculated by the AAA and Monte Carlo simulation using the 6 and 15 MV photon beams, it is concluded that the AAA method underestimated the depth dose in a joint to 10%‐20% when the joint width varied from 2‐0.5 mm. When a bolus of 1.5 cm was added on top of a joint, the AAA method underestimated the depth dose in the joint to 5%‐15%. Moreover, depth doses were found to decrease with an increase of the joint width, according to Monte Carlo results. The AAA method could not predict the change of depth dose with variation of joint width. It is concluded that for the 6 MV photon beams, dependences of joint dose on its width and beam angle were less significant when bolus was added to the phantom. The dosimetry comparison in this study should be useful to radiation staff carrying out treatments where small bone joint is involved as either target or critical tissue in radiotherapy.

## ACKNOWLEDGMENTS

We would like to thank Varti Vartanian and Mitch Spiegel from Varian Medical System for providing detailed information about the 21 EX linear accelerator.

## Supporting information

Supplementary Material
