# Supplementary figures and images for: Dosimetry of small bone joint calculated by the analytical anisotropic algorithm: a Monte Carlo evaluation using the EGSnrc
Source: J Appl Clin Med Phys. 2013 Jan 6;15(1):262–73. doi: 10.1120/jacmp.v15i1.4588 (PMC5711239; doi:10.1120/jacmp.v15i1.4588)

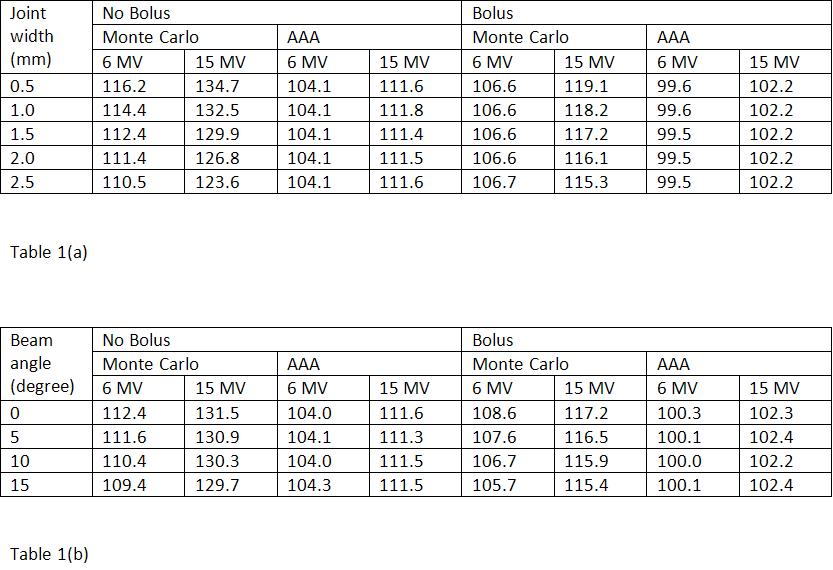

Supplement: Supplementary file 1 — Supplementary Material [file ACM2-15-262-s001.JPG]
